# Decoding vascular calcification-neutrophil signature in pathogenesis of chronic IgA nephropathy: evidence from artificial intelligence-driven multi-omics and *in vitro* validation

**DOI:** 10.3389/fphys.2026.1928397

**Published:** 2026-08-19

**Authors:** Chunyu Cao, Wencheng Yan, Mingyue Li, Jing Lu

**Affiliations:** 1 First Affiliated Hospital of Gannan Medical University, Ganzhou, Jiangxi, China; 2 First Clinical Medical College of Gannan Medical University, Ganzhou, Jiangxi, China

**Keywords:** artificial intelligence, IgA nephropathy, multi-omics, neutrophil, vascular calcification

## Abstract

**Background:**

Dysregulation of neutrophil and consistent vascular calcification(VC) contributes to the pathogenesis and progression of chronic IgA nephropathy(C-IgAN). However, its integrated mechanism in IgAN has not yet been elucidated.

**Methods:**

We integrated GSVA, CIBERSORT and WGCNA algorithms in C-IgAN patient bulk profiles(GSE35487 and GSE93798) downloaded from GEO database for identified VC and neutrophil (VN)-related shared DEGs. Next, explainable and systemic machine learning algorithms in C-IgAN training and validation bulk profiles identified VN-associated diagnostic model and hub gene. In addition, based on VN-associated shared gene signature, we performed NMF analysis for identification of VN-related molecular subgroups, and then we also investigated the molecular and immune patterns between subgroups. Besides, we also investigated hub gene, VC and neutrophil molecular landscapes at C-IgAN patient single-cell level via advanced analytical frameworks, such as sctenifoldknk and Bayes-prism algorithms. Novelty, deep learning algorithm (DrugRefLector) and molecular docking based on C-IgAN identified optimal therapeutic agent targeting hub gene. Finally, *in vitro* assays examined the expression pattern of hub gene.

**Results:**

Our study first traced dynamic VN patterns in C-IgAN and identified VN-associated diagnostic and molecular subgroups. Besides, *CLEC4D* can be considered as neutrophil-distributed hub gene involved in VC and C-IgAN pathogenesis, which also can be considered as a drug target of BRD-K27184429 in C-IgAN.

**Conclusion:**

Our study first identified VN-related patterns for C-IgAN patients, which provides novel idea into clinical applications.

## 1 Introduction

Chronic IgA nephropathy(C-IgAN) is the most common primary glomerulonephritis globally, characterized by the deposition of galactose-deficient IgA immune complexes in the mesangium, leading to progressive renal injury (Rodrigues et al., 2017). The pathogenesis of IgAN involves a complex interplay of genetic susceptibility, mucosal immunity dysregulation, and subsequent inflammatory responses within the kidney (Kamal et al., 2024). Epidemiological studies indicate a higher prevalence in East Asian populations, and it remains a leading cause of chronic kidney disease (CKD) and kidney failure worldwide, imposing a significant healthcare burden (Ghaddar et al., 2024; Cai et al., 2025).

Beyond the classical immune complex-mediated injury, emerging evidence highlights the roles of innate immunity and vascular pathology in IgAN progression (Gesualdo et al., 2021). Neutrophils, as first responders of innate immunity, infiltrate renal tissues in various glomerulonephritis and can release neutrophil extracellular traps (NETs), proteases, and reactive oxygen species, exacerbating tissue damage and inflammation (Zheng et al., 2024; Liu et al., 2025). Vascular calcification (VC), a pathological deposition of calcium-phosphate crystals in the vessel wall, is a common complication in CKD, including IgAN, and is strongly associated with cardiovascular morbidity and mortality (Chen et al., 2023). Importantly, VC in IgAN patients is primarily a consequence of CKD-associated mineral bone disorder (CKD-MBD) and systemic inflammation rather than a primary renal pathology (Siracusa et al., 2024). VC typically develops in the systemic vasculature as renal function declines, and its presence is associated with increased cardiovascular risk in IgAN patients (Siracusa et al., 2024). Interestingly, chronic inflammation, a hallmark of both IgAN and CKD, serves as a critical link between neutrophil activation and the osteogenic transformation of vascular smooth muscle cells that underlies VC (Liu et al., 2025). However, a systematic investigation into the coordinated role of neutrophil dysregulation and VC in the pathogenesis of IgAN, and the identification of key molecular drivers at this intersection, remains lacking.

To address this gap, we conducted an integrative multi-omics study leveraging artificial intelligence (AI), and delineated the infiltration patterns of neutrophils and the activity of VC pathways in IgAN (Ying et al., 2025; Zhou et al., 2025). Besides, we also constructed a VC and neutrophil(VN)-associated molecular subgroup and diagnostic model for IgAN patients. Besides, we further validated its cellular specificity and functional role of hub gene within the renal microenvironment using single-cell RNA sequencing (scRNA-seq) data. Finally, we employed a deep learning-based drug screening approach to nominate a potential therapeutic agent targeting this hub gene, with preliminary validation at *in vitro* studies. This work provides a comprehensive framework linking VN in IgAN, offering novel diagnostic and therapeutic insights.

## 2 Materials and methods

### 2.1 Source of data

Bulk RNA-sequencing datasets were retrieved from the GEO database. GSE35487 (based on GPL96) comprised 6 normal control and 25 IgAN kidney biopsy samples (Reich et al., 2010). GSE93798 (based on GPL22945) contained 6 normal control and 25 IgAN kidney biopsy samples (Liu et al., 2017). GSE116626 (based on GPL14951) included 22 normal and 52 IgAN kidney biopsy samples (Cox et al., 2020). GSE115857 (based on GPL14951) consisted of 7 normal and 55 IgAN kidney biopsy samples (Cox et al., 2020). Batch effects across bulk datasets were assessed using the limma R package (Leek et al., 2012).

### 2.2 WGCNA analysis

The relative abundance of neutrophil infiltration score in GSE35487 was estimated using the CIBERSORT algorithm in R (Chen et al., 2018). The gene set for VC was obtained from the GeneCards database with threshold greater than 1 (Xu et al., 2023). GSVA was performed on GSE93798 using the GSVA package in R for calculating VC score in GSE93798 (Hänzelmann et al., 2013). WGCNA was conducted separately on GSE35487 and GSE93798 using the WGCNA package in R (Langfelder and Horvath, 2008). For each dataset, a soft-thresholding power (β) was selected using the pickSoftThreshold function to achieve a scale-free topology fit index (Langfelder and Horvath, 2008). Modules were identified with a minimum module size of 30 and a merge cut height of 0.25 (Langfelder and Horvath, 2008). Module eigengenes (MEs) were calculated, and modules with the highest absolute Pearson correlation coefficient (|r| > 0.6, p < 0.001) with the neutrophil score (in GSE35487) or the VC GSVA score (in GSE93798) were selected as key modules (Langfelder and Horvath, 2008). All genes within these key modules were extracted for downstream analysis.

### 2.3 Machine learning for diagnostic model construction

A comprehensive machine learning workflow was implemented using the caret and glmnet packages in R (Wang et al., 2022). A total of 132 algorithm combinations were trained on GSE116626 and validated in GSE115857 using 10-fold cross-validation (Wang et al., 2022). The optimal model was selected based on the highest mean area under the receiver operating characteristic curve (AUC-ROC) across cross-validation folds (Wang et al., 2022). The final model, identified as Stemglm(both) + Enet(alpha=0.2), was built using the expression of the 6 shared DEGs (Wang et al., 2022). Its diagnostic performance was evaluated on both the training (GSE116626) and independent validation (GSE115857) sets using AUC-ROC and precision-recall (PR) curves via the pROC package in R (Xu et al., 2023). A nomogram incorporating the six-gene signature was constructed, and its calibration was assessed using a calibration plot with 1000 bootstrap resamples via rms package in R (Xu et al., 2023).

### 2.4 NMF analysis

Based on the expression of the VN-associated shared DEGs, NMF consensus clustering was performed on IgAN samples in GSE116626 using the NMF R package in R (Wang et al., 2024). The optimal number of clusters (k=2) was determined by the cophenetic correlation coefficient (Wang et al., 2024). PCA was used to visualize the separation between the subgroups. Differential immune infiltration between subgroups was analyzed using CIBERSORT package in R (Chen et al., 2018). Gene Set Enrichment Analysis (GSEA) was conducted using the clusterProfiler package in R with the Hallmark gene sets from MSigDB to identify enriched biological pathways (Yu et al., 2012).

### 2.5 Single-cell analysis

The GSE285335(including 11 IgAN patients) in scRNA-seq data was downloaded from GEO database and processed using Seurat package in R (Tan et al., 2023; Kim et al., 2025). Cells with unique feature counts <200 or >6000, or with >20% mitochondrial gene content were filtered out using Seurat package in R (Morabito et al., 2023). Data were normalized using SCTransform, and principal component analysis (PCA) was performed (Morabito et al., 2023). Clustering was done using the FindNeighbors and FindClusters functions (resolution=0.8), resulting in 10 clusters visualized via UMAP and t-SNE using Seurat package in R (Morabito et al., 2023). Cell type annotation was performed using the SingleR package in R (Aran et al., 2019). Cell-cell communication analysis was performed with CellChat package in R (Fang et al., 2022). Metabolic heterogeneity was assessed using SCENITH package in R (Argüello et al., 2020). The AUCell package in R was used to score the activity of the VC gene set in each cell (Chen et al., 2025). Pseudotime trajectory analysis of neutrophils was performed using Monocle2 package in R (Huang et al., 2024). Virtual knockout of the hub gene in neutrophils was simulated using the scTenifoldKnk package in R (Osorio et al., 2022). The top 10 most perturbed genes were subjected to KEGG and GO enrichment analysis using clusterProfiler package in R (Yu et al., 2012). The cellular composition of the bulk tissue in GSE116626 was deconvoluted using the BayesPrism method in R with the annotated scRNA-seq data from GSE285335 as the reference, to estimate neutrophil abundance (Chu et al., 2022).

### 2.6 Drug screening and molecular docking

The deep learning algorithm DrugReflector was applied to the differential expression profile between IgAN and normal samples in GSE116626, referencing the Connectivity Map (cMAP) database to identify compounds predicted to reverse the disease signature (DeMeo et al., 2025). The top candidate compound was selected. Molecular docking between the hub gene-encoded protein and the candidate drug was performed using AutoDock Vina and PyMOL tools (Zhou et al., 2026). The protein structure was retrieved from the PDB database with ID:3WHD and the drug 3D structure with compound CID: 1549000 was obtained from PubChem database (Zhou et al., 2026). The binding affinity was reported in kcal/mol.

### 2.7 *In vitro* validation

To experimentally validate the functional role of *CLEC4D* in neutrophils within the context of IgAN-related VC and inflammation, we designed *in vitro* assays using a neutrophil model system. Given the challenges of transfecting primary human neutrophils, we employed the HL-60 cell line (ATCC,USA), a human promyelocytic leukemia cell line that can be differentiated into N1 neutrophil-like cells. HL-60 cells were cultured in RPMI-1640 medium (Gibco, USA) supplemented with 20% heat-inactivated fetal bovine serum (FBS, Gibco, USA) and 1% penicillin-streptomycin (Gibco, USA). To induce neutrophil differentiation, cells were treated with 1.25% dimethyl sulfoxide (DMSO, Sigma-Aldrich, USA) for 5–7 days. Differentiation was confirmed by assessing morphological changes. N1-like neutrophil was designated as IgAN group and primary neutrophil can be considered as control group. Total RNA was extracted from HL-60 using TRIzol Reagent (Takara, China). cDNA was then synthesized with a PrimeScript RT reagent Kit (Vazyme, China). Quantitative real-time PCR (qRT-PCR) was carried out using TB Green Premix Ex Taq II (Vazyme, China) following the manufacturer’s protocol. The primer sequences (5’→3’) employed in this study are provided below.

### 2.8 Statistical analysis

Group comparisons for continuous variables were performed using Student’s t-test or Mann-Whitney U test, as appropriate. For multiple group comparisons, one-way ANOVA or Kruskal-Wallis test was used, followed by *post-hoc* tests. Correlation analyses used Pearson or Spearman methods. All statistical tests were two-sided, and p < 0.05 was considered significant. Analyses were performed in R.

## 3 Results

### 3.1 Identification of neutrophil-associated patterns for IgAN patients

**Figure 1:**
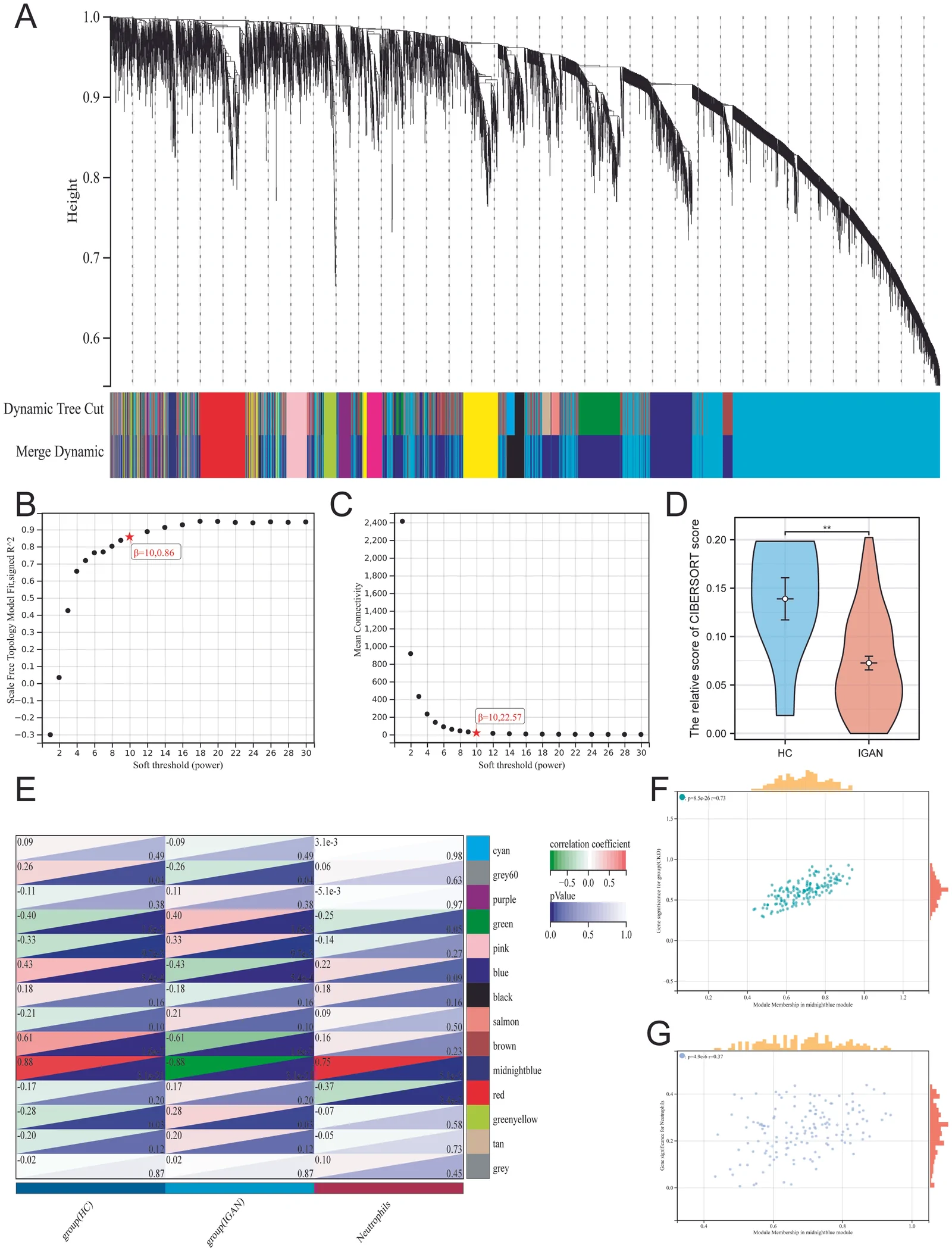
Identification of neutrophil-associated correlated module for IgAN patients. **(A)** Cluster dendrogram of genes and module colors identified by WGCNA. **(B, C)** Cluster dendrogram of genes and module colors identified by WGCNA in GSE35487. **(D)** Box plot comparing the neutrophil infiltration score between normal and IgAN groups. **(E)** Heatmap of the correlation between module eigengenes (MEs) and the neutrophil score. **(F, G)** Scatter plot of gene significance (GS) for midnightblue. ** means p < 0.01.

To investigate the role of innate immunity and vascular pathology in IgAN, we first analyzed immune cell infiltration using the CIBERSORT algorithm on dataset GSE35487. We observed a significant reduction in neutrophil abundance in IgAN kidney biopsies compared to normal controls (**Figure 1D**). To explore the molecular network associated with this dysregulation, we performed WGCNA (**Figure 1A**). A scale-free topology was achieved with a soft-thresholding power of β = 10 (**Figures 1B, C**). The resulting ‘midnightblue’ module showed the strongest positive correlation with the neutrophil score (r=0.75, p=1e-05), and high module membership of its constituent genes confirmed their central role in neutrophil-associated biology (**Figures 1E–G**). We finally extracted the genes in this module.

### 3.2 Identification of VC-associated patterns for IgAN patients

**Figure 2:**
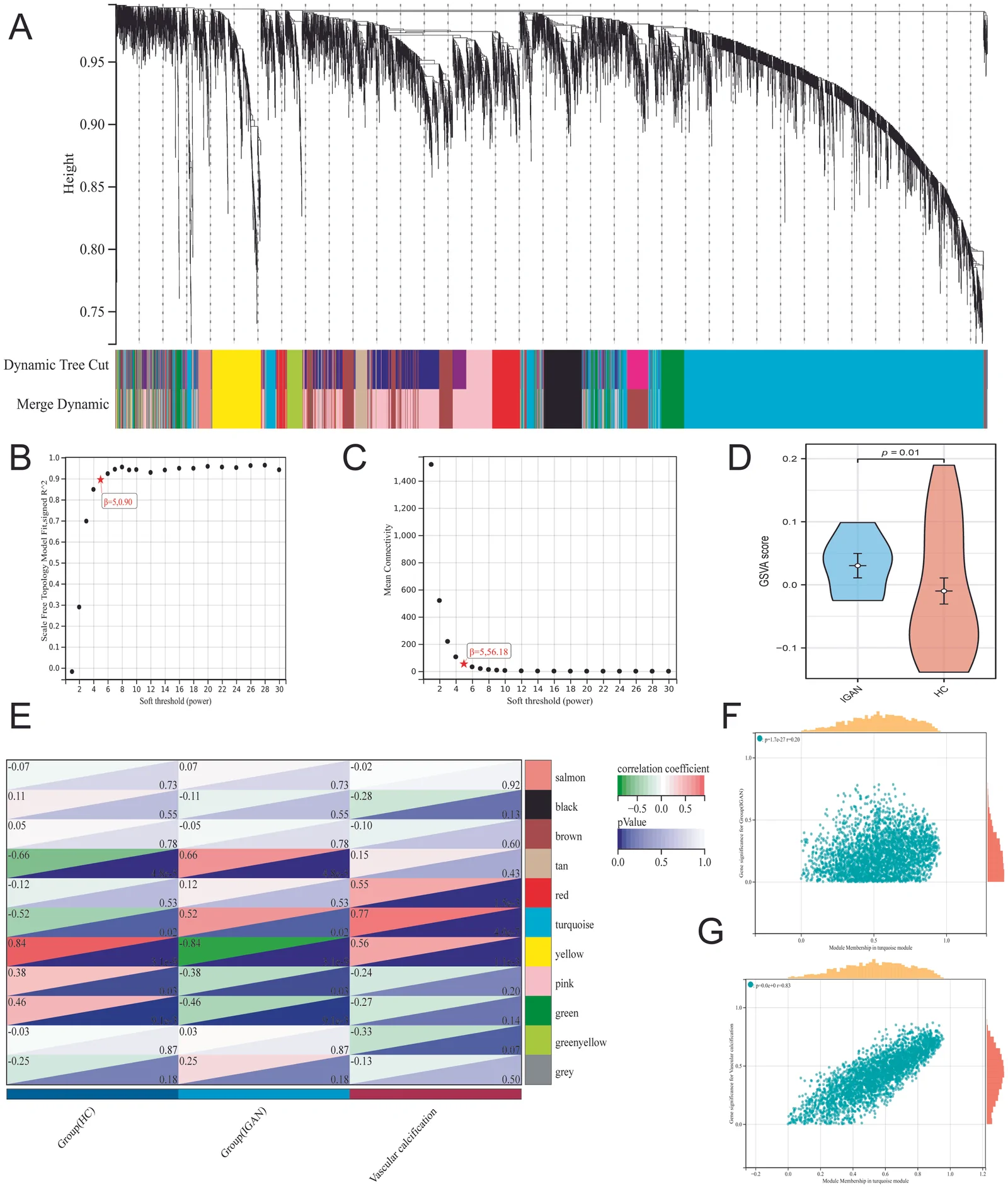
Identification of VC-associated correlated module for IgAN patients. **(A)** Cluster dendrogram of genes and module colors identified by WGCNA. **(B, C)** Cluster dendrogram of genes and module colors identified by WGCNA in GSE93798. **(D)** Box plot comparing the neutrophil infiltration score between normal and IgAN groups. **(E)** Heatmap of the correlation between module eigengenes (MEs) and the neutrophil score. **(F, G)** Scatter plot of gene significance (GS) for midnightblue.

In parallel, we assessed VC pathway activity in dataset GSE93798 (internal set 2) using GSVA. The VC score was significantly downregulated in IgAN samples (**Figure 2D**). WGCNA on this dataset (soft-threshold β = 5) identified a ‘turquoise’ module with a strong negative correlation to the VC score (r=-0.77, p=4.0e-7), indicating its genes are inversely related to VC activity (**Figures 2A–G**). Finally, we extracted the genes in this module.

### 3.3 VN-associated diagnostic model construction for IgAN patients

**Figure 3:**
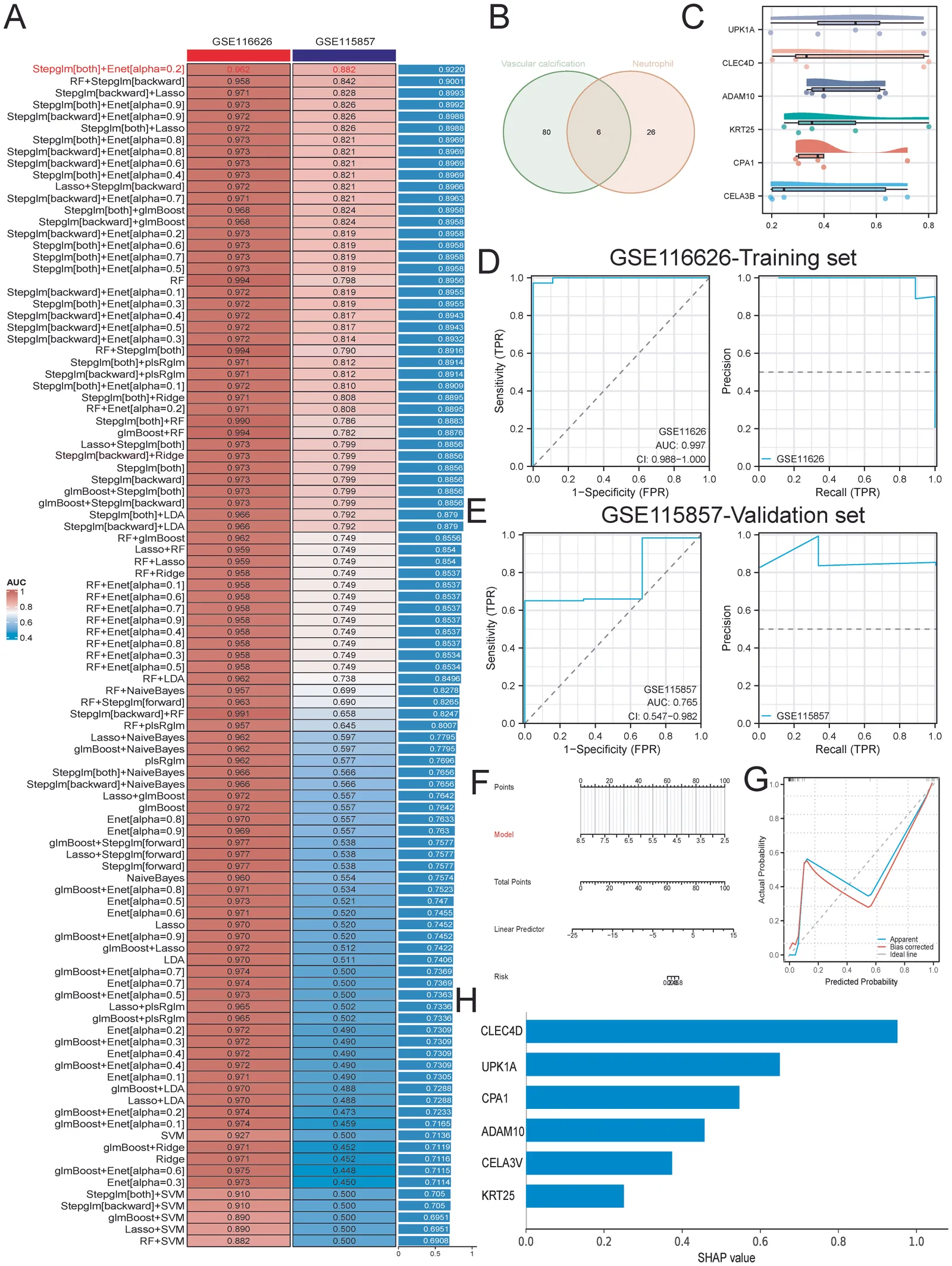
Construction and validation of a VN diagnostic model for IgAN patients. **(A)** Performance metrics (mean AUC-ROC) of the machine learning algorithm combinations during cross-validation on the training set and validation set. **(B)** Venn diagram showing the intersection of genes from the neutrophil-related module and the VC-related module, yielding 6 shared genes (*UPK1A*, *CLEC4D*, *ADAM10*, *KRT25*, *CPA1*, *CELA3B*). **(C)** Friend analysis of 6 shared genes (*UPK1A*, *CLEC4D*, *ADAM10*, *KRT25*, *CPA1*, *CELA3B*). **(D, E)** Model estimation of in GSE116626 and GSE115857. **(F, G)** Calibration and nomogram plot, showing good agreement between predicted and observed probabilities. **(H)** SHAP summary plot illustrating the contribution of each gene to the model output.

We intersected genes from the neutrophil-correlated module and the VC-correlated module, yielding 6 shared genes: *UPK1A*, *CLEC4D*, *ADAM10*, *KRT25*, *CPA1*, and *CELA3B* (**Figures 3B, C**). To evaluate their diagnostic potential, we employed a systematic machine learning framework on the training set GSE116626. After training 132 algorithm combinations, the Stemglm(both) + Enet(alpha=0.2) model demonstrated the highest and most stable performance during cross-validation (mean AUC = 0.9220) (**Figure 3A**). This six-gene model achieved excellent diagnostic accuracy on both the training set (AUC = 0.997) and an independent validation set GSE115857 (AUC = 0.765) (**Figures 3D, E**). Precision-Recall curves further confirmed its robust performance (**Figures 3D, E**). A nomogram integrating the expression of these 6 genes provided a practical tool for probability estimation, with a calibration plot indicating strong agreement between predicted and observed outcomes (**Figures 3F, G**). To interpret the model, SHAP analysis was performed, which identified *CLEC4D* as the feature with the greatest mean absolute contribution to the model predictions, establishing it as the central hub gene (**Figure 3H**).

### 3.4 VN-associated molecular subgroups for IgAN patients

**Figure 4:**
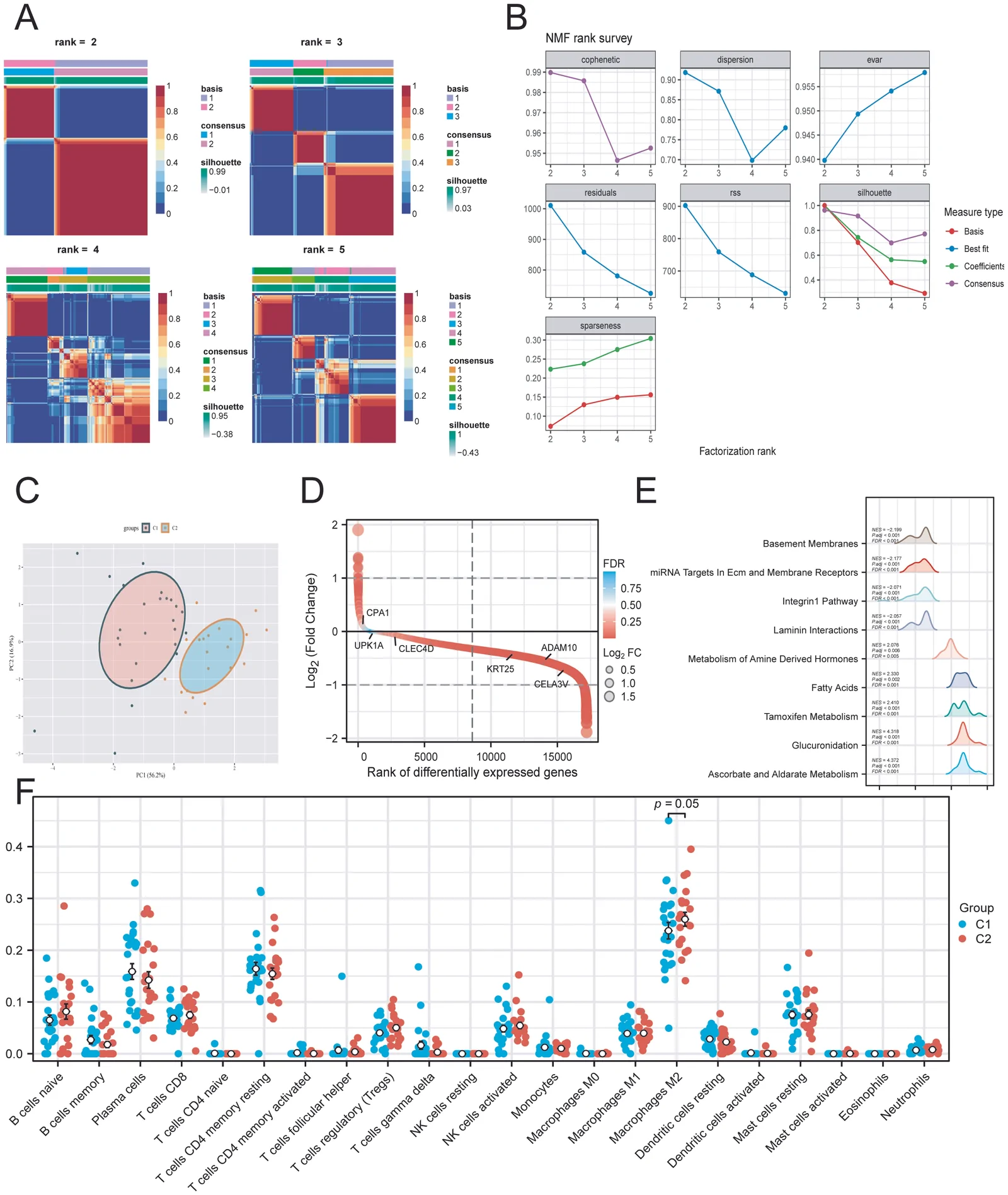
Identification and characterization of VN-related molecular subgroups in IgAN patients. **(A, B)** NMF analysis. **(C)** PCA plot showing clear separation between the two subgroups. **(D)** Box plots comparing the expression levels of the 6 signature genes between C1 and C2. **(E)** GSEA enrichment plots between C1 and C2. **(F)** Heatmap of differential immune cell infiltration (CIBERSORT scores) between C1 and C2.

We next investigated whether the 6-gene vascular calcification-neutrophil (VN) signature could reveal heterogeneity among IgAN patients (**Figure 4A**). Non-negative matrix factorization (NMF) consensus clustering based on this signature in the GSE116626 cohort robustly classified patients into two subgroups: Cluster 1 (C1, n=32) and Cluster 2 (C2, n=20) (**Figure 4B**), with clear separation on a PCA plot (**Figure 4C**). Differential expression analysis confirmed significant variations in the signature genes between the 2 clusters (**Figure 4D**). Immune deconvolution analysis revealed that C2 was characterized by significantly higher infiltration of macrophage M2 compared to C1 (**Figure 4F**). Besides, GSEA analysis between C1 and C2 was used for estimation of molecular heterogeneity (**Figure 4E**).

### 3.5 Single-cell atlas of IgAN patients

**Figure 5:**
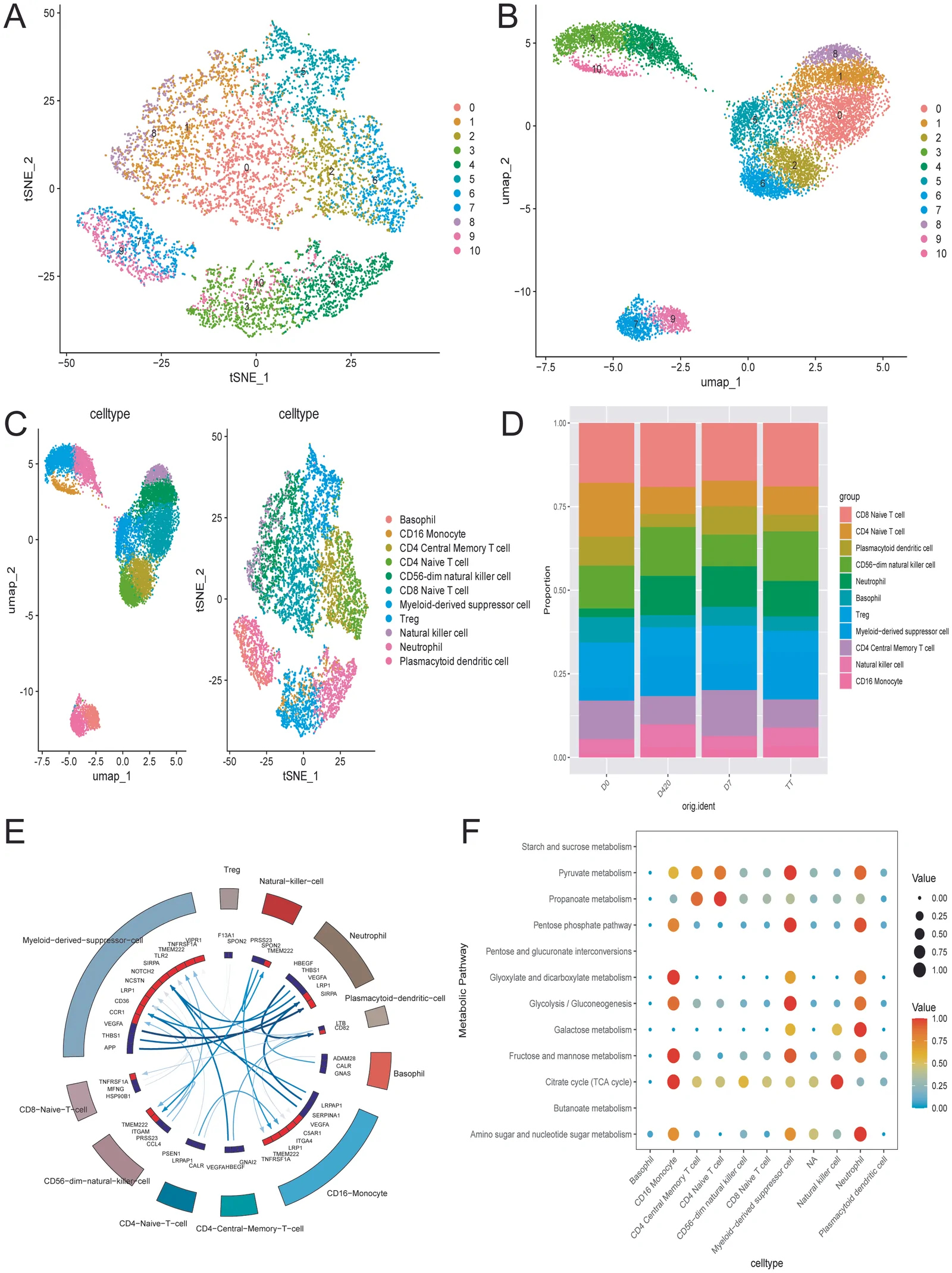
Single-cell atlas of IgAN patients. **(A, B)** UMAP and t-SNE visualization of 10 cell clusters. **(C)** UMAP and t-SNE visualization of annotated with 11 major cell types using SingleR. **(D)** Proportion of each cell type across all samples. **(E)** CellChat analysis results showing the number and strength of inferred interactions between cell types. **(F)** Heatmap of metabolic pathway activity across cell types analyzed by scMetabolism.

To validate our findings at a cellular level, we analyzed scRNA-seq data from IgAN kidneys (GSE285335). After quality control and clustering, we annotated 10 cell clusters and 11 major cell types (**Figures 5A–D**). CellChat analysis highlighted neutrophils as active participants in intercellular communication, mainly interacting with myeloid-derived suppressor cell and plasmacytoid dendritic cell (**Figure 5E**). Next, the metabolic heterogeneity among these cell types were analyzed (**Figure 5F**).

### 3.6 Molecular patterns of VN-associated hub gene for IgAN patients

**Figure 6:**
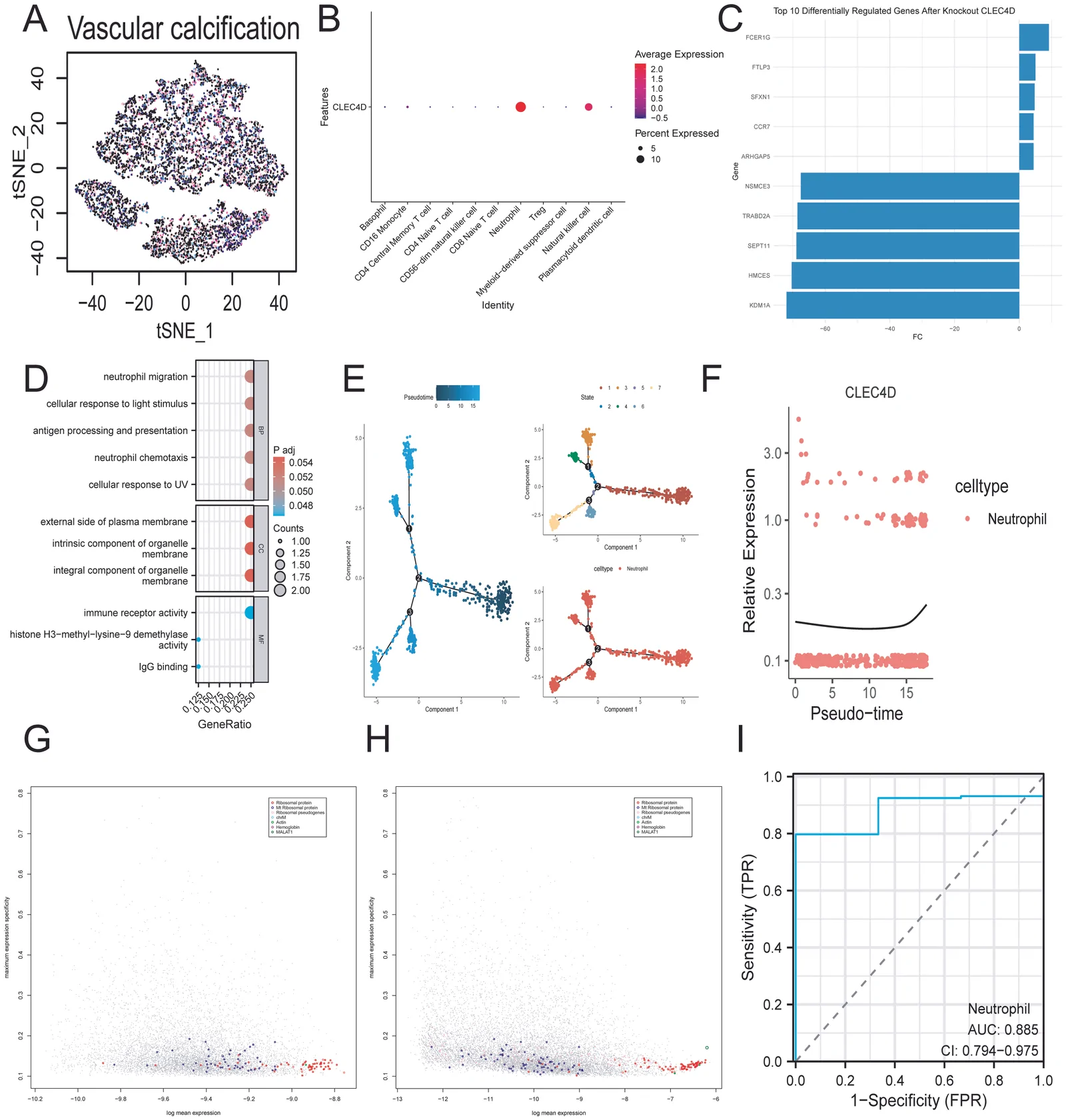
Functional dynamics of *CLEC4D* in IgAN neutrophils. **(A)** AUCELL of VC at single-cell level. **(B)** Expression of *CLEC4D* at single-cell level. **(C)** Virtual knockout (KO) of *CLEC4D* in neutrophils using scTenifoldKnk. **(D)** KEGG and GO enrichment analysis of perturbed genes. **(E, F)** Monocle2 analysis of neutrophil and *CLEC4D*. **(G–I)** Scatter plot showing the correlation between deconvoluted neutrophil abundance from BayesPrism and the *CLEC4D* expression level in bulk IgAN samples from GSE116626.

It can be witnessed that VC-associated signature was mainly activated in neutrophil (**Figure 6A**). Remarkably, feature plot and violin plot analyses demonstrated that *CLEC4D* expression was almost exclusively restricted to the neutrophil cluster (**Figure 6B**). Furthermore, scoring the VC gene set activity (AUCell) across cell types revealed that the VC pathway was most active precisely within these *CLEC4D*+ neutrophils (**Figure 6A**). Pseudotime trajectory analysis of neutrophils identified 6 differentiation states, with *CLEC4D* expression showing a clear increasing trend along the differentiation path (**Figures 6E, F**). To infer *CLEC4D* function, we performed an *in silico* knockout using scTenifoldKnk, which perturbed a network of genes in neutrophils (**Figure 6C**). Enrichment analysis of these perturbed genes linked them to key neutrophil functions such as neutrophil migration and immune response (**Figure 6D**). Finally, using Bayesian deconvolution (BayesPrism), we projected the neutrophil signature from the scRNA-seq data onto the bulk samples of GSE116626, confirming a significant positive correlation between estimated neutrophil abundance and *CLEC4D* expression in IgAN patients (**Figures 6G–I**).

### 3.7 Therapeutic screening for IgAN patients and clinical validation

**Figure 7:**
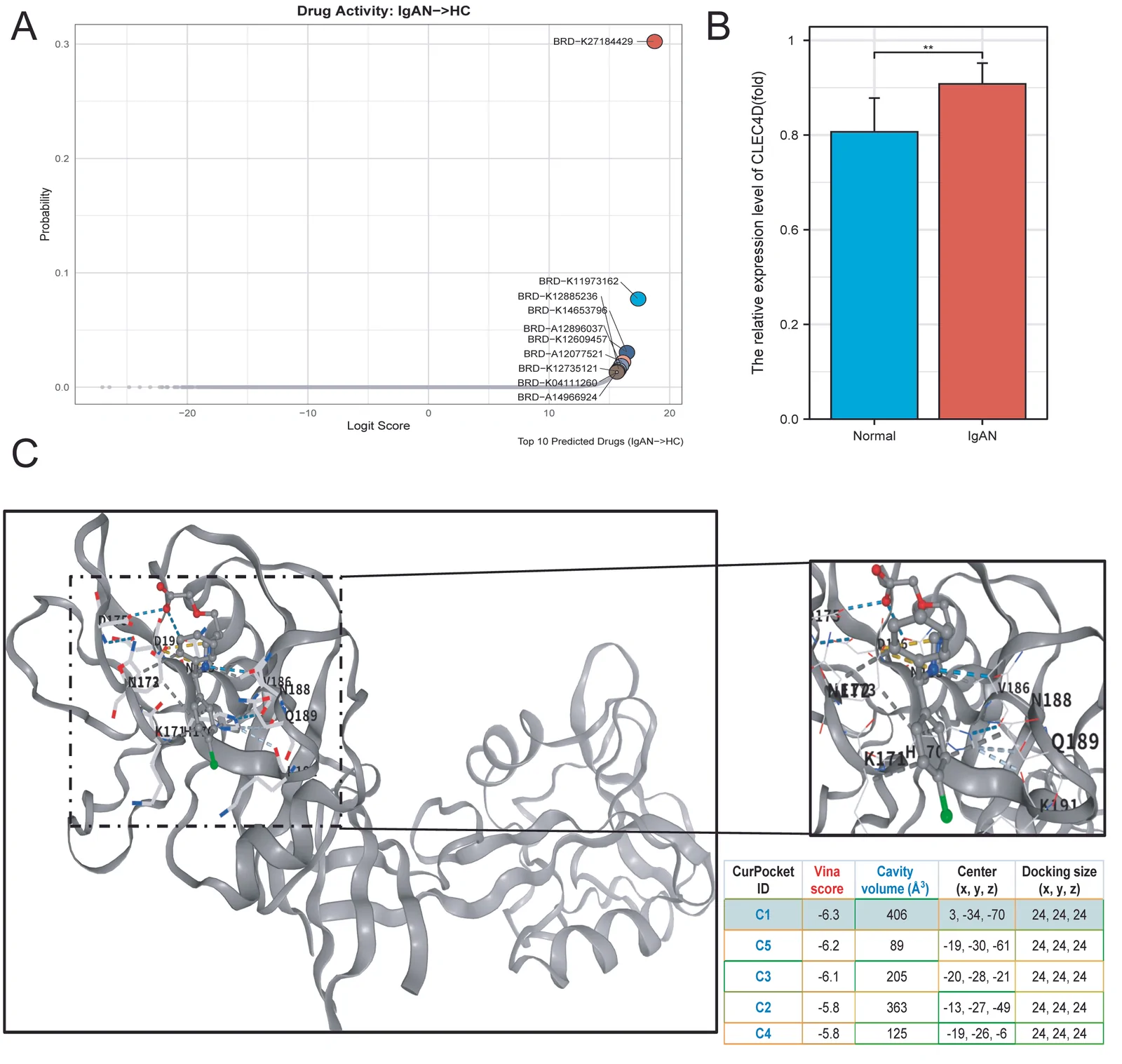
AI-driven drug discovery and preliminary clinical validation. **(A)** Workflow of DrugReflector analysis applied to the IgAN signature from GSE116626 against the cMAP database. **(B)** q-RT-PCR validation of *CLEC4D* mRNA expression. **(C)** Schematic of molecular docking between the *CLEC4D* protein and BRD-K27184429. ** means p < 0.01.

To translate our findings into therapeutic potential, we applied the deep learning algorithm DrugReflector to the disease signature from GSE116626, screening the cMAP database for compounds predicted to reverse it (**Figure 7A**). This analysis nominated BRD-K27184429 as the top candidate agent (**Figure 7A**). Molecular docking simulations predicted a strong and stable interaction between BRD-K27184429 and the *CLEC4D* protein, with a favorable binding affinity of -6.3 kcal/mol (**Figure 7C**). As an initial step towards clinical relevance, we measured *CLEC4D* mRNA levels at *in vitro* assays. Consistent with our bioinformatics findings, *CLEC4D* expression was significantly upregulated in IgAN patients (**Figure 7B**).

## 4 Conclusion and discussion

Our integrated multi-omics study systematically deciphers the coordinated role of vascular calcification (VC) and neutrophil dysregulation in the pathogenesis of IgAN. We identified a robust six-gene signature (*UPK1A*, *CLEC4D*, *ADAM10*, *KRT25*, *CPA1*, *CELA3B*) at the intersection of these two processes, which demonstrated high diagnostic accuracy. Through explainable AI, *CLEC4D* emerged as the central hub gene. Notably, this signature stratified IgAN patients into 2 distinct molecular subgroups with divergent inflammatory and immune profiles, suggesting its potential for patient classification.

*CLEC4D* (C-type lectin domain family 4 member D) is a pattern recognition receptor primarily expressed on myeloid cells, known to recognize fungal components and modulate immune responses (Yang et al., 2024). Previous studies have reported *CLEC4D* involvement in various inflammatory conditions (Zhang et al., 2025). In gastric cancer, *CLEC4D* has been shown to promote proliferation and migration via the NF-κB/AKT signaling pathway (Yang et al., 2024). In sarcopenia, knockdown of *CLEC4D* alleviates myoblast dysfunction and inflammation by inhibiting the JAK/STAT pathway (Ma et al., 2025). While *CLEC4D* has not been extensively studied in kidney diseases, one prior study suggested a genetic association of *CLEC4D* with systemic sclerosis-related interstitial lung disease (Luo et al., 2023). However, its functional role in IgAN pathogenesis has been entirely unexplored until now. Our study is the first to implicate *CLEC4D* in IgAN pathophysiology. In neutrophils, it can influence adhesion, migration, and the release of inflammatory mediators (Ma et al., 2025). Chronic inflammation driven by activated neutrophils is a key driver of VC in IgAN (Stoneman et al., 2026). Neutrophil-derived enzymes like myeloperoxidase and matrix metalloproteinases can directly damage vascular tissue and promote an osteogenic phenotype in vascular smooth muscle cells (Li et al., 2025). We hypothesize that *CLEC4D*, by potentiating neutrophil activation and recruitment within the renal and systemic vasculature, may exacerbate the inflammatory milieu that fosters VC. Our single-cell analysis strongly supports this, placing *CLEC4D* expression and high VC pathway activity specifically within kidney-infiltrating neutrophils. The virtual knockout experiment further linked *CLEC4D* to genes regulating neutrophil migration and immune activation. While *CLEC4D* has been studied in infections and other inflammatory conditions, its role in renal disease, particularly IgAN, is novel (Ma et al., 2025). One prior study suggested a genetic association of *CLEC4D* with lupus nephritis, but its functional role in IgAN pathogenesis has been entirely unexplored until now (Luo et al., 2023). Our findings position *CLEC4D* as a novel molecular nexus linking innate immune dysregulation and vascular pathology in IgAN.

In conclusion, this study provides the first comprehensive multi-omics landscape integrating vascular calcification and neutrophil signatures in IgAN. We identified and validated *CLEC4D* as a neutrophil-specific hub gene within this axis, developed a diagnostic model, and nominated a potential therapeutic agent. These findings illuminate a previously underappreciated pathogenic pathway in IgAN and lay the groundwork for developing novel diagnostic and therapeutic strategies targeting the neutrophil-VC nexus. However, the interpretative scope of this study is bound by several limitations. The foundational transcriptomic insights are derived from publicly available datasets, which, despite batch correction, may not fully account for all sources of technical and biological variability. Future studies should combine the clinical information of C-IgAN patients, focusing on the estimation of efficacy of diagnostic and molecular stratification models acquired from our study in large, multi-center clinical trials to enhance clinical significance. Besides, the single-cell analysis, while offering high-resolution insights, is based on a cohort of 11 C-IgAN patients, potentially limiting the capture of the full heterogeneity of neutrophil states across the C-IgAN spectrum. Future studies should combined advanced AI technologies in higher resolution single-cell and spatial data of C-IgAN patients, coupled with pre-clinical studies, dynamically tracing VC-associated gene signature in neutrophil for the pathogenesis of C-IgAN patients. In addition, the precise molecular cascades downstream of *CLEC4D* in C-IgAN in neutrophil that ultimately impair renal health via regulating VC remain to be fully delineated in pre-clinical C-IgAN models. Furthermore, Future research should prioritize *in vivo* studies using conditional knockout or neutrophil-specific overexpression of *CLEC4D* in C-IgAN mouse models to establish causality and elucidate detailed mechanisms acquired by our *in silico* knockout. Concurrently, high-throughput screening for neutrophil-selective *CL4C4D* modulators could yield more targeted therapeutic leads than existing pan-inhibitors. Similarly, the therapeutic candidate, BRD-K27184429, identified computationally, demands rigorous experimental testing in preclinical models to confirm its efficacy, optimal dosing, and ability to penetrate the blood-brain barrier and modulate the proposed VN-associated axis and *CLEC4D* *in vivo*.

## Data Availability

The datasets presented in this study can be found in online repositories. The names of the repository/repositories and accession number(s) can be found in the article/**Supplementary Material**.
