# Supplementary material for: Decoding vascular calcification-neutrophil signature in pathogenesis of chronic IgA nephropathy: evidence from artificial intelligence-driven multi-omics and in vitro validation
Source: Front Physiol. 2026 Aug 19;17:1928397. doi: 10.3389/fphys.2026.1928397 (PMC13533995; doi:10.3389/fphys.2026.1928397)
Supplement: Supplementary file 1 [file SupplementaryFile1.docx]

**Figure S1: Pre-processing of single-cell data. (A-C)** QC metrics of single-cell. **(D)** Heatmap of cell clusters.
